# Assessment of Stromal Calretinin Expression in Normal and Pathological Endometrium of Patients With Abnormal Uterine Bleeding

**DOI:** 10.7759/cureus.89854

**Published:** 2025-08-12

**Authors:** Taj Mohd, Pooja Jaiswal, Priyanka Singh, Nausheen S Khan, Lovely Singh, Syed F Mustaqueem

**Affiliations:** 1 Pathology, Integral Institute of Medical Sciences and Research, Lucknow, IND

**Keywords:** abnormal uterine bleeding, calretinin, endometrial carcinoma, endometrial hyperplasia, endometrial stroma, immunohistochemistry

## Abstract

Background: Abnormal uterine bleeding (AUB) is a prevalent gynecological issue, often arising from structural or functional endometrial abnormalities. Calretinin, a calcium-binding protein, has demonstrated diagnostic relevance in various tissues; however, its role in endometrial stroma remains insufficiently explored. This study was conducted to evaluate calretinin expression in normal and pathological endometrial tissues associated with AUB.

Materials and methods: A prospective observational study was conducted on 60 endometrial samples (normal and AUB-associated lesions) over two years from June 2023 to March 2025. Histopathological evaluation was followed by immunohistochemistry for stromal calretinin using a semi-quantitative score (0-5+). Statistical analysis was performed using IBM SPSS Statistics v25.0 (IBM Corp., Armonk, USA), including analysis of variance (ANOVA), Tukey's post hoc test, and Pearson's correlation.

Results: Normal endometrium (notably secretory and proliferative phases) exhibited higher stromal calretinin expression (mean scores: 3.0 and 2.0, respectively), while lesions such as adenomyosis, endometrial polyps, hyperplasia, and carcinoma demonstrated markedly reduced or absent expression. A statistically significant difference in calretinin scores was found across different histopathological categories (p=0.0002). A moderate negative correlation (r=-0.43) was observed between calretinin expression and pathological endometrial states.

Conclusion: Stromal calretinin expression was substantially reduced in AUB-related endometrial lesions compared to normal endometrium. These findings suggest its potential utility as a supplementary biomarker for distinguishing between physiological and pathological endometrial conditions. Further large-scale studies are warranted to validate its diagnostic and prognostic significance.

## Introduction

Abnormal uterine bleeding (AUB) is a common gynecological condition characterized by alterations in menstrual timing, volume, or duration, often indicating underlying hormonal, endometrial, or structural disorders. AUB can manifest acutely as sudden-onset or excessively heavy menstrual bleeding, often requiring urgent medical intervention, or as a chronic condition persisting for six months or longer, necessitating structured evaluation and long-term management [1-3]. The International Federation of Gynecology and Obstetrics (FIGO) classifies AUB etiologies using the PALM-COEIN system: structural causes include polyps, adenomyosis, leiomyoma, and malignancy/hyperplasia (PALM), while non-structural causes encompass coagulopathies, ovulatory dysfunction, endometrial causes, iatrogenic factors, and unclassified entities (COEIN) [4].

AUB affects approximately 10-30% of women of reproductive age and up to 50% of perimenopausal women and is a major contributor to anemia, fatigue, psychological stress, and diminished quality of life [5-7]. The underlying cause often varies with age: ovulatory and endometrial dysfunction (AUB-O and AUB-E) are more common in younger women, whereas structural abnormalities like fibroids (AUB-L) and malignancies (AUB-M) predominate in older, postmenopausal women [3]. Hence, histopathological evaluation of endometrial biopsies is crucial for identifying lesions that range from benign polyps and hyperplasia to endometrial carcinoma. The endometrial stroma responsive to hormonal signaling plays a vital role in endometrial remodeling and may provide a diagnostic of AUB [8].

Calretinin, a 29 kDa calcium-binding protein belonging to the EF-hand family, is encoded by the CALB2 gene and has well-established roles in calcium homeostasis, cell signaling, and apoptosis [9]. Though classically utilized as an immunohistochemical (IHC) marker in mesotheliomas and ovarian sex cord-stromal tumors [10], its presence in the endometrial stroma has drawn recent interest. Calretinin expression is known to fluctuate with hormonal phases, showing strong, diffuse cytoplasmic positivity in stromal cells during the functional phase and declining in postmenopausal, hyperplastic, or neoplastic conditions [11,12]. A study by Al Moghrabi et al. (2007) demonstrated this zonal stromal staining pattern in normal endometrium and its loss in pathological states like dysfunctional uterine bleeding (DUB), endometrial polyps, and carcinoma [11]. Similarly, Mai et al. (2008) observed diminished calretinin expression in DUB and noted “disordered stromal architecture” with basal layer stromal cells extending into the functional layer, detected through combined calretinin and CD34 immunostaining [13]. In addition to its diagnostic relevance, calretinin has shown potential functional implications in reproductive tissues. It is expressed in ovarian thecal and stromal cells, corpus luteum, and secretory endometrial stroma, likely regulated by hormonal modulators such as 1α,25-dihydroxyvitamin D3 [12,14]. While its role in mesothelial and neuroendocrine tissues is well established, stromal calretinin expression in the endometrium remains underexplored, particularly in the context of AUB.

Calretinin has emerged as a promising stromal marker due to its distinct zonal expression pattern in hormonally responsive endometrial stroma. Prior studies have demonstrated strong cytoplasmic calretinin staining in functional endometrium, with reduced or absent expression in pathological conditions such as DUB, endometrial hyperplasia, and endometrial carcinoma. This downregulation is thought to reflect alterations in stromal differentiation and hormonal responsiveness, making calretinin a potentially useful marker in distinguishing between physiological and pathological endometrial changes [11,13].

Based on current evidence, stromal calretinin shows distinct expression patterns between normal and pathological endometrium. However, comprehensive studies quantitatively evaluating calretinin expression across specific AUB-associated lesions are limited. Additionally, the role of histological features such as stromal glandular dissociation, a condition characterized by the separation or disorganization of endometrial stromal and glandular elements, is not well established in relation to calretinin expression. Therefore, the present study aimed to: (i) evaluate stromal calretinin expression in both normal and abnormal endometrial tissue associated with AUB; (ii) correlate expression patterns with histopathological subtypes; and (iii) explore the association of calretinin expression with clinical parameters such as age and lesion type.

## Materials and methods

Study design and setting

This observational study was conducted in the Department of Pathology at Integral Institute of Medical Sciences and Research (IIMSR), Lucknow, Uttar Pradesh, India, over a two-year period from June 2023 to March 2025. The study included endometrial samples obtained via biopsy or hysterectomy from patients presenting with AUB to the Department of Obstetrics and Gynecology. Tissue specimens were collected in 10% buffered neutral formalin and subjected to routine histopathological and IHC evaluation. All the samples included in the study were specimens of normal endometrium and endometrial lesions from patients with AUB. Patients with a history of postmenopausal hormone replacement therapy were excluded from the study. The study was approved by the Institutional Ethics Committee (approval number: IEC/IIMSR/2023/05, dated 16th May 2023). A written informed consent was obtained from each participant.

Sample collection and histopathological processing

A total of 60 formalin-fixed paraffin-embedded (FFPE) tissue samples were included. The samples comprised both normal endometrium (across proliferative and secretory phases) and AUB-associated lesions, such as hyperplasia, polyps, disordered proliferative endometrium, and malignancies. Tissues were processed following standard protocols for paraffin embedding. Sections of 3-4 µm thickness were prepared and stained with hematoxylin and eosin (H&E) for histopathological evaluation under light microscopy. Histopathological diagnoses were assigned according to established criteria: proliferative and secretory endometrium; disordered proliferative endometrium; endometrial hyperplasia with and without atypia (per World Health Organization (WHO) Endometrial Intraepithelial Neoplasia (EIN) guidelines); benign polyps; endometritis; and endometrial carcinoma subtypes (endometrioid, serous, clear cell) [15,16]. Although all samples were collected from patients presenting with AUB, a subset of cases demonstrated histologically normal endometrium (proliferative, secretory, or menstrual phase) on H&E staining. These were included in the “normal” category as incidental findings and served as internal comparators to AUB-associated pathological lesions. Demographic and clinical information, including patient age, presenting symptoms, menstrual history, and clinical indication for biopsy, were obtained from the hospital’s electronic medical records and pathology requisition forms. Where necessary, additional relevant clinical data were verified through patient interviews conducted at the time of biopsy or sample collection. All data were anonymized prior to analysis.

IHC staining for calretinin

FFPE tissue sections of 3-4 µm thickness were mounted on poly-L-lysine-coated slides and incubated at 56°C for one hour. Deparaffinization was performed in xylene, followed by rehydration through graded alcohols. Antigen retrieval was carried out using Tris(hydroxymethyl)aminomethane-ethylenediaminetetraacetic acid (Tris-EDTA) buffer (pH 9.0) in a pressure cooker at 95°C for 30 minutes. Slides were allowed to cool naturally at room temperature for 20 minutes before proceeding. Endogenous peroxidase activity was blocked by incubating the slides in 3% hydrogen peroxide for 10 minutes. Non-specific binding was blocked using a casein-based protein block (for 10 minutes at room temperature). The primary monoclonal mouse anti-calretinin antibody (clone H-5, Quartett, Germany) was applied at a dilution of 1:100, as per the manufacturer's datasheet, and incubated for one hour at room temperature. This was followed by application of a post-primary reagent and a horseradish peroxidase (HRP)-conjugated polymer secondary antibody (Quartett, Germany), each incubated for 20 minutes. Slides were rinsed in phosphate-buffered saline (PBS) between each step. Visualization was achieved using 3,3′-diaminobenzidine (DAB) chromogen for five minutes, followed by counterstaining with Mayer’s hematoxylin. Slides were dehydrated, cleared, and mounted. Appropriate positive (mesothelium) and negative controls were included with each staining run.

Evaluation of IHC expression

**Figure 1:**
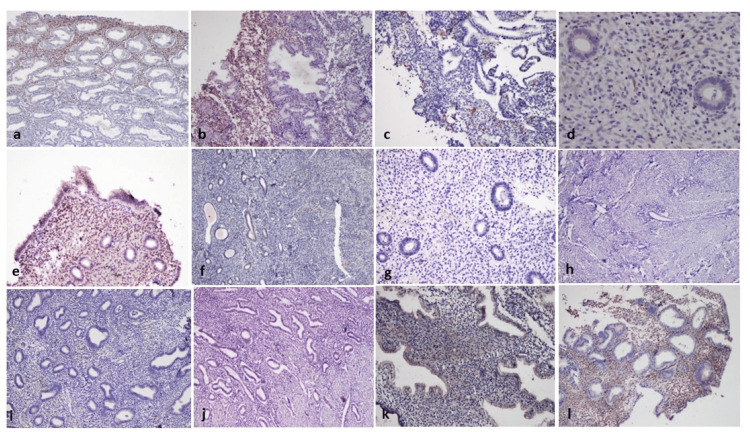
Representative microphotographs showing calretinin IHC staining in various endometrial histopathological conditions (a) Strong positive expression in proliferative endometrium (IHC, 10×). (b) Strong positive expression in secretory endometrium (IHC, 20×). (c) Focal positivity in menstrual endometrium (IHC, 20×). (d) Focal expression in proliferative endometrium in AUB case (IHC, 40×). (e) Strong expression in secretory endometrium in AUB case (IHC, 20×). (f) Focal positivity in atrophic endometrium (IHC, 10×). (g) Negative expression in chronic endometritis (IHC, 20×). (h) Negative expression in adenomyosis (IHC, 10×). (i) Negative expression in endometrial hyperplasia without atypia (IHC, 20×). (j) Negative expression in atypical endometrial hyperplasia (IHC, 20×). (k) Focal positivity in secretory endometrium with progestational effect (IHC, 40×). (l) Strong positivity in stromal-glandular dissociation (IHC, 10×). AUB: Abnormal uterine bleeding; IHC: Immunohistochemical

Calretinin IHC staining in stromal cells was evaluated semi-quantitatively by a panel of experienced pathologists. The evaluation was based on the percentage of stromal cells exhibiting cytoplasmic positivity, using a scoring system. Each tissue section was carefully examined under high-power fields, and only cytoplasmic staining within stromal cells was considered as positive immunoreactivity. The scoring was categorized into six levels based on the proportion of positive cells observed: Score 0 indicated negative staining or less than 1% positivity; Score 1 corresponded to 1-5% positive cells; Score 2 was assigned for 6-10% positivity; Score 3 reflected 11-30% positive stromal cells; Score 4 included cases with 31-60% positive cells; and Score 5 denoted more than 60% stromal cells showing cytoplasmic positivity. This stratified scoring approach allowed for a reproducible and objective assessment of calretinin expression across the study samples. This scoring method reflects commonly accepted practices for calretinin IHC evaluation [11,17]. Epithelial staining, if present, was not evaluated in this study (Figures 1a-1l). All slides were independently evaluated by two experienced pathologists who were blinded to the clinical and histopathological diagnoses. This blinding was implemented to minimize observer bias during the interpretation of IHC staining. Discrepancies in scoring were resolved by joint review and consensus. Although interobserver agreement was not quantified using statistical methods such as the kappa coefficient, consensus-based evaluation has been widely used in similar IHC studies and ensures consistent interpretation.

Statistical analysis

Data were entered and analyzed using Microsoft Excel (Microsoft Corp., Redmond, USA) and IBM SPSS Statistics v25.0 (IBM Corp., Armonk, USA). Descriptive statistics were used to summarize demographic and clinicopathological variables. Mean and standard deviation (SD) were calculated for the dataset. For comparison of calretinin expression intensity across different histopathological diagnoses, one-way analysis of variance (ANOVA) was performed, followed by Tukey’s Honestly Significant Difference (HSD) post hoc test to identify pairwise group differences. Correlation between calretinin expression and categories such as normal endometrium and AUB was analyzed using Pearson’s correlation coefficient (r). A p-value <0.05 was considered statistically significant for all comparisons.

## Results

Frequency distribution of endometrial lesions across age groups

A total of 60 endometrial cases were evaluated in the present study, including AUB-associated lesions and normal endometrium. The frequency distribution of various histopathological diagnoses across age groups is summarized in Table 1. Among the AUB cases, atrophic endometrium exhibited the highest mean age of presentation (55.7 years), seen predominantly in women aged 51-66 years. Endometrioid adenocarcinoma was found in the age group of 48-49 years, with a mean age of 48.5 years. On the other hand, chronic endometritis and secretory endometrium were observed in younger women (mean ages 32.0 and 33.4 years, respectively). Cases of proliferative and secretory endometrium, both in normal and AUB groups, were distributed across a wide age range but were most frequently encountered in the 31-40 years age group. Endometrial hyperplasia without atypia and stromal glandular dissociation peaked in the 41-50 years group. Adenomyosis was largely seen in the 31-50 years age range. Notably, endometrial polyps and chronic endometritis were more common among women aged 21-40 years. The normal endometrial phases (menstrual, proliferative, and secretory) had a mean age range of 35.0 to 40.8 years.

**Table 1:** Frequency distribution of endometrial lesions across age groups AUB: Abnormal uterine bleeding

Category	Histopathological Diagnosis	Number (n)	Mean Age (yrs)	Min Age	Max Age
Normal endometrium	Menstrual endometrium	2	40.0	40	40
	Proliferative endometrium	5	40.8	30	45
	Secretory endometrium	5	35.0	32	40
AUB cases	Adenomyosis	5	44.8	38	60
	Atrophic endometrium	6	55.7	43	66
	Atypical endometrial hyperplasia	1	35.0	35	35
	Chronic endometritis	3	32.0	24	40
	Endometrial hyperplasia (no atypia)	5	40.6	29	50
	Endometrial polyp	5	36.2	30	45
	Endometrioid adenocarcinoma	2	48.5	48	49
	Proliferative endometrium	5	35.2	28	40
	Secretory endometrium	5	33.4	28	45
	Secretory endometrium (progestational effect)	5	39.0	35	44
	Stromal glandular dissociation	6	39.5	30	46

Distribution of calretinin reactivity across histopathological diagnoses

**Figure 2:**
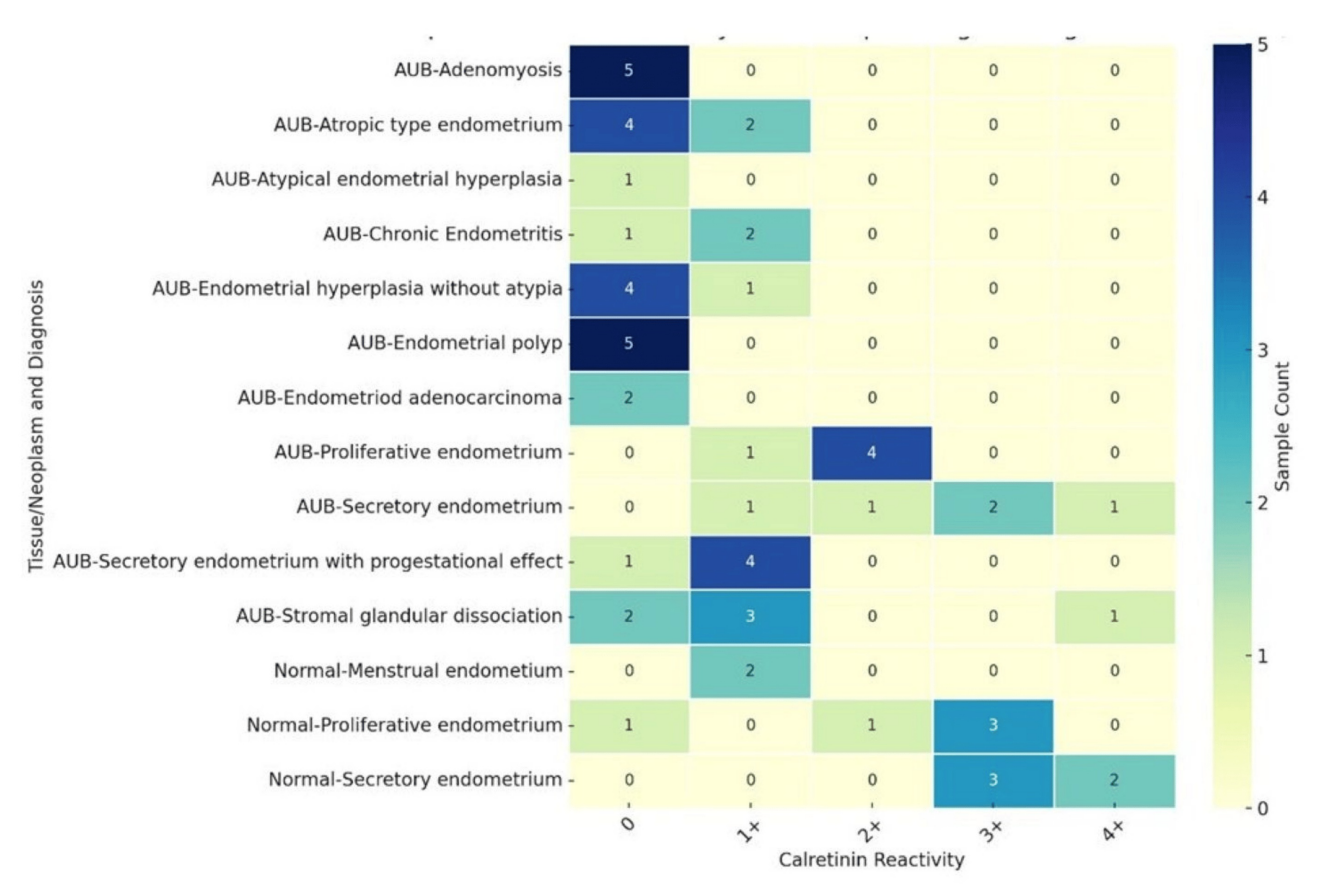
Heatmap showing distribution of calretinin immunoreactivity across histopathological diagnoses and tissue types in endometrial lesions The heatmap illustrates the frequency of calretinin reactivity scores (ranging from 0 to 4+) across various combinations of tissue types (normal and AUB) and histopathological diagnoses. Each row represents a unique pairing of tissue/neoplasm type with its histological diagnosis, while columns show the degree of calretinin expression. The color intensity corresponds to the number of cases exhibiting each reactivity level. Most normal endometrial tissues showed moderate to strong calretinin reactivity, while AUB-associated lesions predominantly exhibited weak or absent staining. AUB: Abnormal uterine bleeding

The IHC evaluation of calretinin expression across different histopathological categories revealed significant variation between normal endometrium and AUB cases, as presented in Table 2 and Figure 2. Among the normal endometrial samples, all cases of menstrual endometrium showed low calretinin expression (1+), with no negative cases. In the proliferative endometrium, one case was negative, while the others showed moderate to high expression (0-3+). Secretory endometrium demonstrated consistent expression (ranging from 3 to 4+), indicating robust stromal calretinin presence in functional endometrial phases. In contrast, a majority of AUB-associated lesions displayed reduced or absent calretinin expression. Adenomyosis, endometrial hyperplasia without atypia, endometrial polyps, and atrophic endometrium exhibited a high number of negative cases (4-5 out of 5-6 samples), suggesting diminished calretinin presence in these structural or degenerative conditions. Endometrioid adenocarcinoma cases (n=2) were entirely negative for calretinin, indicating a complete loss of stromal expression in malignant tissue. Chronic endometritis and secretory endometrium with progestational effect showed minimal expression (0-1+). However, some AUB cases, particularly those with proliferative and secretory endometrial histology, retained calretinin positivity (1-4+), similar to their normal counterparts. Stromal glandular dissociation displayed variable expression ranging from negative to moderate (0-4+), reflecting heterogeneity in its stromal component.

**Table 2:** Frequency distribution of calretinin reactivity across histopathological diagnoses AUB: Abnormal uterine bleeding

Category	Tissue/Neoplasm	Negative (n)	Calretinin Reactivity
Normal endometrium	Menstrual endometrium	0	1
	Proliferative endometrium	1	0-3
	Secretory endometrium	0	3-4
AUB cases	Adenomyosis	5	0
	Atrophic endometrium	4	0-1
	Atypical endometrial hyperplasia	1	0
	Chronic endometritis	1	0-1
	Endometrial hyperplasia (no atypia)	5	0
	Endometrial polyp	5	0
	Endometrioid adenocarcinoma	2	0
	Proliferative endometrium	0	1-2
	Secretory endometrium	0	1-4
	Secretory endometrium (progestational effect)	1	0-1
	Stromal glandular dissociation	2	0-4

Comparison of calretinin expression across histopathological diagnoses

The IHC analysis of calretinin expression across various histopathological categories revealed a heterogeneous staining pattern, suggesting an association between marker expression and the physiological or pathological nature of endometrial tissue, as depicted in Table 3. Uniform negativity for calretinin was observed in adenomyosis, endometrial polyp, and endometrial hyperplasia without atypia, where all respective cases (n=5 each) showed no immunoreactivity. Similarly, both cases of endometrioid adenocarcinoma demonstrated complete absence of calretinin staining, although the small sample size limits broader interpretation. Atrophic endometrium (n=6) exhibited a mixed expression profile: two cases showed weak staining (1+), while four were negative, consistent with the reduced proliferative capacity in atrophic tissue. Chronic endometritis (n=3) demonstrated weak immunoreactivity (1+) in two-thirds of cases, with one negative case. Atypical endometrial hyperplasia (n=1) was completely negative. Distinctively, normal cycling endometrial phases exhibited more pronounced and varied expression. Proliferative endometrium (n=10) showed a wide range of staining intensities: one case with weak positivity (1+), five cases with moderate staining (2+), three cases with strong positivity (3+), and one negative case. Secretory endometrium (n=10) exhibited relatively higher staining intensities, with five cases showing strong positivity (3+), three cases with very strong positivity (4+), and the remaining showing weaker expression (1+ and 2+). These findings suggest increased calretinin expression associated with functional endometrial changes during the menstrual cycle. Secretory endometrium with progestational effect (n=5) demonstrated predominantly weak expression: four cases showed 1+ positivity, while one case was negative, possibly indicating reduced expression due to exogenous hormonal influence. Stromal glandular dissociation (n=6) displayed variable expression, with three cases showing 1+, one case showing strong positivity (4+), and two negative cases, reflecting the histopathological heterogeneity of the lesion. Menstrual endometrium (n=2) exhibited uniformly weak positivity (1+), supporting baseline marker presence during the shedding phase.

**Table 3:** Comparison of calretinin expression across histopathological diagnoses ANOVA: Analysis of variance

Histopathological Diagnosis	1+	2+	3+	4+	Negative
Adenomyosis	0	0	0	0	5
Atrophic type endometrium	2	0	0	0	4
Atypical endometrial hyperplasia	0	0	0	0	1
Chronic endometritis	2	0	0	0	1
Endometrial hyperplasia (no atypia)	0	0	0	0	5
Endometrial polyp	0	0	0	0	5
Endometrioid adenocarcinoma	0	0	0	0	2
Menstrual endometrium	2	0	0	0	0
Proliferative endometrium	1	5	3	0	1
Secretory endometrium	1	1	5	3	0
Secretory endometrium (progestational effect)	4	0	0	0	1
Stromal glandular dissociation	3	0	0	1	2
ANOVA F-statistic: 10.39, P-value: 0.0002					

Mean calretinin expression intensity across histopathological diagnoses

**Figure 3:**
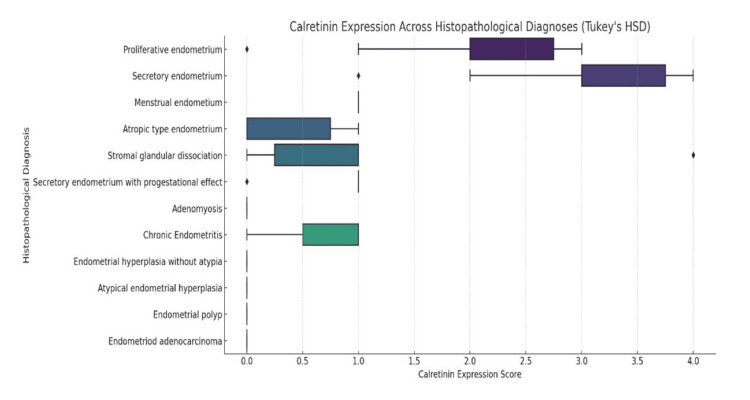
Box plot showing comparison of mean calretinin expression across different histopathological subtypes Tukey’s HSD: Tukey’s Honestly Significant Difference

The mean calretinin expression intensity across various histopathological diagnoses is presented in Figure 3. The highest mean expression was observed in secretory endometrium (mean=3.0), followed by proliferative endometrium (mean=2.0), indicating strong stromal calretinin positivity in these physiological phases. Moderate expression was observed in stromal glandular dissociation (mean=1.17) and menstrual endometrium (mean=1.0). Secretory endometrium with progestational effect demonstrated lower expression (mean=0.80). Minimal or no expression (mean=0.0) was observed in adenomyosis, endometrial polyp, endometrioid adenocarcinoma, and endometrial hyperplasia without atypia. Statistical analysis using one-way ANOVA revealed a highly significant difference in mean calretinin expression across the different histopathological categories (p=0.0002). Subsequent Tukey’s HSD post hoc analysis identified that significant differences exist between secretory endometrium vs. stromal glandular dissociation (p=0.0022) and secretory endometrium vs. secretory endometrium (progestational effect) (p=0.0003) (Table 4). Adenomyosis, endometrial polyp, and hyperplasia without atypia showed no significant differences, confirming that these conditions exhibit similar calretinin expression levels.

**Table 4:** Tukey’s HSD post hoc analysis of differences between histological diagnoses Tukey’s HSD: Tukey’s Honestly Significant Difference

Histopathology Group 1	Histopathology Group 2	Mean Difference	P-value	Significant
Adenomyosis	Atrophic type endometrium	0.33	0.999	No
Adenomyosis	Atypical endometrial hyperplasia	0.0	1.0	No
Adenomyosis	Chronic endometritis	0.67	0.989	No
Adenomyosis	Endometrial hyperplasia (no atypia)	0.0	1.0	No
Adenomyosis	Endometrial polyp	0.0	1.0	No
Proliferative endometrium	Secretory endometrium (progestational effect)	-1.20	0.217	No
Proliferative endometrium	Stromal glandular dissociation	-0.83	0.655	No
Secretory endometrium	Secretory endometrium (progestational effect)	-2.20	0.0003	Yes
Secretory endometrium	Stromal glandular dissociation	-1.83	0.0022	Yes
Secretory endometrium (progestational effect)	Stromal glandular dissociation	0.37	0.999	No

Correlation between calretinin expression and endometrial categories

To assess the relationship between calretinin expression and endometrial categories, Pearson’s correlation analysis was conducted, as presented in Table 5. A moderate negative correlation was observed between calretinin expression and normal endometrium (r=-0.435), indicating that higher calretinin expression tends to be associated with a lower frequency of normal endometrial cases. Similarly, a moderate negative correlation was found between calretinin expression and AUB cases (r=-0.438), suggesting reduced calretinin expression in pathological endometrial conditions such as adenomyosis, endometrial hyperplasia, and endometrial polyps.

**Table 5:** Correlation between calretinin expression and endometrial categories AUB: Abnormal uterine bleeding

Variable Pair	Correlation Coefficient (r)	Interpretation
Normal endometrium vs. calretinin expression	-0.435	Moderate inverse correlation
AUB cases vs. calretinin expression	-0.438	Moderate inverse correlation

## Discussion

There is currently no consensus regarding the effectiveness of calretinin as a biomarker for differentiating between benign, hyperplastic, and malignant endometrial conditions. To fill this knowledge gap, the present study systematically evaluated calretinin expression in normal endometrial stroma and its alterations in various endometrial lesions related to AUB. By correlating calretinin levels with histopathological characteristics, age distribution, and other clinicopathological variables, this research aimed to shed light on the possible diagnostic and prognostic implications of calretinin in the context of endometrial pathology.

In the present study, we examined the distribution of various histopathological diagnoses related to endometrial lesions in cases of normal endometrium and AUB. We also assessed the mean, minimum, and maximum ages at which these conditions occur. The average age for normal endometrial categories was found to be between 35 and 40.8 years. In contrast, AUB cases exhibited a wider age distribution (24-66 years), with atrophic endometrium identified in the oldest demographic (mean age: 55.7 years). Conditions such as adenomyosis (mean age: 44.8 years) and endometrioid adenocarcinoma (mean age: 48.5 years) were predominantly found in middle-aged women. Benign hyperplastic lesions, including endometrial polyps (mean age: 36.2 years) and endometrial hyperplasia without atypia (mean age: 40.6 years), were more common among younger women. Chronic endometritis had the lowest mean age (32 years, range 24-40), indicating its prevalence in younger women of reproductive age.

Previous research indicates that proliferative and secretory endometria are predominantly observed in women of reproductive age, specifically between 20 and 40 years, which aligns with our findings of a mean age ranging from 35 to 40.8 years [18]. Additionally, adenomyosis is often reported in perimenopausal women aged 40 to 50 years, consistent with the current study's mean age of 44.8 years [19]. Endometrial hyperplasia and polyps are prevalent among women in their late reproductive and perimenopausal years (35 to 45 years), corroborated by the mean ages observed in this research (36.2 to 40.6 years) [20]. Endometrioid adenocarcinoma is typically diagnosed in postmenopausal women aged 50 to 60 years; however, the current study indicates a slightly younger mean age of 48.5 years, suggesting a potential trend toward earlier onset that warrants further investigation [21]. Atrophic endometrium in patients with AUB is generally associated with postmenopausal bleeding, with literature indicating a peak age of 55 to 65 years, which is consistent with the mean age of 55.7 years found in the current study [22]. While the current findings are in agreement with existing literature, they also reveal certain discrepancies, such as the earlier age of presentation in some cases, like endometrioid adenocarcinoma, which may point to regional, genetic, or environmental factors. The findings emphasize the critical need for age-appropriate screening and management strategies for AUB to guarantee timely diagnosis and intervention.

This study identified a broad array of histopathological lesions associated with AUB. The most frequently observed lesions were endometrial hyperplasia, polyps, chronic endometritis, adenomyosis, and endometrioid adenocarcinoma. These findings corroborate previous research that indicates structural abnormalities, hormonal imbalances, and chronic inflammatory processes are significant contributors to AUB [23]. A key observation from this research is the age-related variation in the prevalence of these lesions. Younger women were primarily found to exhibit proliferative and hyperplastic changes, while older women showed a higher incidence of atrophic endometrium, adenomyosis, and malignancies. This reinforces the notion that hormonal fluctuations and ongoing estrogenic stimulation may be integral to the development of diverse endometrial lesions that lead to AUB [24]. This research contributes to a more comprehensive understanding of the pathophysiological foundations of AUB and the potential role of calretinin in the remodeling of the endometrium. If further studies validate these results, calretinin may emerge as an important supplementary biomarker in the histopathological analysis of endometrial lesions, facilitating the distinction between benign and malignant conditions.

In the healthy endometrium, calretinin expression was noted in the stromal cells, particularly during the proliferative and secretory phases, with varying intensities. The presence of calretinin in the normal endometrial stroma implies its involvement in processes such as endometrial growth, differentiation, and cyclical remodeling [11]. However, the exact function of calretinin remains unclear, highlighting the need for further investigation to determine its biological role. A major finding of the present study was the significant reduction of calretinin expression in pathological endometrial conditions compared to normal stroma. Specifically, adenomyosis and endometrial hyperplasia cases showed little to no calretinin expression, indicating a potential loss of its role in maintaining stromal balance. Chronic endometritis and atrophic endometrium displayed low but inconsistent expression levels, suggesting a possible relationship between inflammatory processes and calretinin downregulation. Additionally, endometrial polyps and endometrioid adenocarcinoma exhibited minimal or absent calretinin expression, supporting the notion that the loss of calretinin may contribute to uncontrolled endometrial proliferation and tumor development [25].

The results of this study are consistent with earlier research wherein calretinin exhibited limited expression in normal endometrial stroma and a progressive decline in pathological states [11]. The presence of calretinin in normal endometrial stroma corroborates findings that highlight its involvement in stromal remodeling throughout the menstrual cycle, especially during the proliferative phase (with a reactivity score of 2-3 in this investigation) [11]. The absence of calretinin in conditions such as adenomyosis, endometrial hyperplasia, and endometrial polyps aligns with existing literature that notes its lack in benign stromal proliferations [13]. The minimal expression observed in endometrioid adenocarcinoma (scoring 0-1) is noteworthy, as prior studies indicate a downregulation of stromal calretinin in malignant cases, which may be associated with a loss of differentiation and tumor advancement [26]. The heightened expression in secretory endometrium under the influence of progestational factors supports research suggesting that hormonal fluctuations, particularly progesterone dominance, may affect calretinin levels in the stroma. These findings imply that calretinin could be a valuable marker for distinguishing between normal and pathological endometrial tissues, particularly in hyperplastic and neoplastic scenarios. The absence of calretinin in adenomyosis, hyperplasia, and polyps underscores its significance in normal stromal function and suggests its potential role in the development of lesions associated with AUB. The weak expression in endometrioid adenocarcinoma indicates that calretinin may not serve as a dependable diagnostic marker for endometrial cancers; however, its absence could assist in differentiating between certain benign and malignant conditions [27]. The pronounced expression in secretory endometrium influenced by progestational factors may reflect a hormonal regulation of calretinin, warranting further investigation into its role in hormone-driven changes within the endometrium.

The statistical analysis demonstrated a moderate inverse correlation (-0.43) between calretinin expression and cases of AUB, indicating that a reduction in calretinin levels correlates with an increased likelihood of pathological endometrial conditions associated with AUB. Further examination of clinicopathological parameters revealed that calretinin expression varied according to histopathological type and patient age. The inverse relationship between calretinin expression and endometrial lesions in older patients suggests that age-related changes in stromal function may lead to lower calretinin levels, thereby predisposing these patients to abnormal endometrial changes. This finding highlights the potential role of calretinin as a marker for assessing stromal integrity and pathological transformation.

Studies indicate that calretinin is expressed at moderate to strong levels in normal proliferative and secretory endometrium, with its highest expression noted during the secretory phase. This is consistent with our findings, where secretory endometrium typically showed 3+ and 4+ expression [11]. The observed decline in calretinin during menstrual shedding further supports previous research that highlights the downregulation of stromal markers during the menstrual phase, which corresponds with our detection of only weak (1+) expression in the menstrual endometrium [13].

The expression of calretinin in lesions associated with adenomyosis and endometrial polyps has been observed to be minimal or absent, likely due to changes in stromal differentiation, which aligns with the findings of the current study, where all cases tested negative for calretinin. Literature consistently reports that endometrioid adenocarcinoma does not express calretinin, corroborating our results of complete negativity in both instances [28]. This indicates that calretinin may not serve as a reliable stromal marker in cases of malignant transformation. In contrast, chronic endometritis and atrophic endometrium displayed some weak positivity (1+), a phenomenon previously documented in inflammatory endometrial conditions characterized by residual stromal activity. Additionally, stromal glandular dissociation exhibited weak positivity (1+ and 4+ in select cases), supporting previous research that highlights the heterogeneous behaviour of stroma in glandular-stromal interactions [13]. The pronounced calretinin expression (4+) observed in secretory endometrium under progestational influence may suggest a hormonal regulatory mechanism, an area that remains under-researched and warrants further exploration. The total absence of calretinin in cases of endometrial hyperplasia and adenomyosis reinforces the notion that calretinin is downregulated in pathological stromal proliferations [7]. These observations imply that calretinin expression is associated with normal stromal activity in the endometrium, peaking during the secretory phase [29]. The loss of calretinin in endometrial polyps, hyperplasia, adenomyosis, and carcinoma indicates its potential role as a marker of normal stromal function, with downregulation occurring in pathological states [7]. Its complete absence in adenomyosis and carcinoma suggests that calretinin is not implicated in their pathogenesis, rendering it an unlikely candidate for diagnostic purposes in these lesions. There is a need for more comprehensive research on the hormonal effects on calretinin expression in the secretory and progestational endometrium, which could be significant for elucidating the hormone-mediated changes occurring in the endometrial stroma.

The significant changes in calretinin expression across various endometrial lesions suggest its potential role as an adjunct marker in the histopathological evaluation of endometrial biopsies [7]. Its absence in hyperplastic and malignant conditions indicates that it may help differentiate benign lesions from pre-malignant and malignant ones. If validated through larger studies, the downregulation of calretinin could reflect a shift toward an abnormal endometrial microenvironment, potentially serving as a predictive marker for disease progression in AUB cases. Exploring the functional significance of calretinin in maintaining stromal homeostasis could lead to innovative targeted therapies that aim to restore normal stromal function in patients with AUB and associated endometrial disorders.

This study is limited by a relatively small sample size, especially within certain histopathological subgroups, which may reduce statistical power and generalizability. The absence of functional assays restricts the interpretation of the biological role of calretinin in endometrial remodeling. Additionally, the single-center design and histopathology-based inclusion may limit the generalizability of findings to broader populations. The lack of hormonal receptor correlation (e.g., estrogen receptor (ER)/progesterone receptor (PR)) and the absence of longitudinal follow-up data further restricts insight into hormonal modulation and temporal dynamics of calretinin expression. Future multicenter studies incorporating hormonal receptor profiling and prospective follow-up are needed to validate these findings and clarify the biomarker's clinical relevance.

## Conclusions

This study demonstrated that stromal calretinin expression varies significantly across different endometrial histopathological categories. Strong expression was observed in physiological endometrial phases, particularly the secretory and proliferative phases, while pathological lesions such as adenomyosis, endometrial polyps, hyperplasia, and carcinoma showed reduced or absent expression. These findings suggest that calretinin may serve as a supportive marker in differentiating normal from abnormal endometrial conditions. However, due to the study’s single-center design and limited sample size, further large-scale, multi-institutional studies are warranted to confirm the diagnostic and prognostic utility of calretinin in routine endometrial pathology.
